# Seed Endophyte bacteria enhance drought stress tolerance in *Hordeum vulgare* by regulating, physiological characteristics, antioxidants and minerals uptake

**DOI:** 10.3389/fpls.2022.980046

**Published:** 2022-10-05

**Authors:** Zainul Abideen, Massimiliano Cardinale, Faisal Zulfiqar, Hans-Werner Koyro, Sarwat Ghulam Rasool, Kamel Hessini, Walid Darbali, Fengliang Zhao, Kadambot H.M. Siddique

**Affiliations:** ^1^ Dr. Muhammad Ajmal Khan Institute of Sustainable Halophyte Utilization, University of Karachi, Karachi, Pakistan; ^2^ Institute of Plant Ecology, Research Centre for Bio Systems, Land Use, and Nutrition (IFZ), Justus-Liebig-University Giessen, Giessen, Germany; ^3^ Institute of Applied Microbiology, Research Centre for Bio Systems, Land Use, and Nutrition (IFZ), Justus-Liebig-University Giessen, Giessen, Germany; ^4^ Department of Biological and Environmental Sciences and Technologies (DiSTeBa), University of Salento, Lecce, Italy; ^5^ Department of Horticultural Sciences, Faculty of Agriculture and Environment, The Islamia University of Bahawalpur, Bahawalpur, Pakistan; ^6^ Department of Biology, College of Sciences, Taif University, Taif, Saudi Arabia; ^7^ Environment and Plant Protection Institute, Chinese Academy of Tropical Agricultural Science (CATAS), Haikou, China; ^8^ The UWA Institute of Agriculture, The University of Western Australia, Perth, WA, Australia

**Keywords:** bacterial inoculation, ecophysiology, endophyte (DSE), oxidative stress, photosynthesis

## Abstract

Growth stimulating bacteria help remediate dry arid soil and plant stress. Here, *Pseudomonas* sp. and *Pantoea* sp. we used to study the stress ecology of *Hordeum vulgare* and the environmental impact of water deficit on soil characteristics, growth, photosynthesis apparatus, mineral acquisition and antioxidiant defense. Plants inoculated with *Pseudomonas* or *Pantoea* had significantly higher (about 2 folds) soil carbon flux (soil respiration), chlorophyll levels (18%), net photosynthetic rate (33% in *Pantoea* and 54% in *Pseudomonas*), (44%) stomatal conductance than uninoculated plants in stressed conditions. Both bacterial strains improved leaf growth (23-29%) and root development under well-watered conditions but reduced around (25%) root biomass under drought. Plants inoculated with *Pseudomonas* or *Pantoea* under drought also increased of about 27% leaf respiration and transpiration (48%) but decreased water use efficiency, photoinhibition (91%), and the risk of oxidative stress (ETR/A) (49%). Drought stress increased most of the studied antioxidant enzymatic activities in the plants inoculated with *Pseudomonas* or *Pantoea*, which reduce the membrane damage and protect plants form oxidative defenses. Drought stress increased K^+^ acquisition around 50% in both shoots inoculated with *Pseudomonas* or *Pantoea* relative to non-stressed plants. Plants inoculated with *Pseudomonas* or *Pantoea* increased shoot Na^+^ while root Na^+^ only increased in plants inoculated with *Pseudomonas* in stressed conditions. Drought stress increased shoot Mg^2+^ in plants inoculated with *Pseudomonas* or *Pantoea* but did not affect Ca^2+^ relative to non-stressed plants. Drought stress increased about 70% K^+^/Na^+^ ratio only in plants inoculated with *Pseudomonas* relative to non-stressed plants. Our results indicate that inoculating barley with the studied bacterial strains increases plant biomass and can therefore play a role in the environmental remediation of drylands for food production.

## Introduction

Irregular climate changes, the increasing population, and intensive agriculture are directly connected to land degradation and food shortages, inducing extreme weather events and environmental impacts in many countries (Munir et al., 2021). Increasing agricultural production, food security and protecting water reserves are critical for sustainable agriculture and environmental safety. Decreased water supply due to declining rainfall affects biological systems, nutrient supply, and crop productivity (Peña-Gallardo et al., 2019; Tyagi and Pandey, 2022). For example, barley (*Hordeum vulgare* L.) biomass and grain yield have substantially declined in arid regions. Limited food crop productivity is associated with reduced water and nutrient flux, causing significant economic losses and socio-economic issues (Kour and Yadav, 2022). New emerging agricultural techniques are acquired to overcome land degradation and increase crop biomass production (Pittelkow et al., 2015; Siddiqui et al., 2021). One strategy is to explore endophytic bacteria, which establish a symbiotic relationship with the host plant and synthesis of nutrients that offer favorable conditions to resist water stress in plants (Rahman et al., 2018; Kour and Yadav, 2022).

Microbial biotechnology is a promising approach for increasing edible plant biomass under stress conditions (Cardoso et al., 2018; Goudarzi et al., 2023). Microbial supplementation can significantly promote bioremediation, control phytopathogens, and increase plant physiological performance and productivity on degraded arid lands (Oleńska et al., 2020). Soil microbial supplementation is influenced by root exudates that produce different enzymes and metabolites, nutrient accumulation, and hormone production (Singh and Gupta, 2018; Zayed et al., 2022). Plant growth-promoting bacteria (PGPB) improve growth and can protect plants against biotic and abiotic stresses by producing volatile compounds, siderophores, growth hormones, biological nitrogen fixation, and reducing plant ethylene synthesis (Khatoon et al., 2020; Das et al., 2022). Identifying the signaling pathways governing the associations between plants and different PGPB can play an important role in improving agricultural production sustainably (Wiggins et al., 2022).

Changes in root development or leaf elongation can be modulated under drought stress but are closely associated with leaf metabolic status and growth portioning (Knutzen et al., 2015; Abideen et al., 2021). Plant-available nutrients (K^+^, Ca^2+^, Mg^2+^ and N) and carbon metablosim with water accessibility through osmotic balance are important mechanisms of plants for photosynthesis and leaf metabolites production under drought (Abdelaal et al., 2021b). However, the ecophysiological responses of plants to reach a new homeostasis after PGPB inoculation under water deficit are not well understood. Inoculation with PGPB could facilitate water uptake, protecting leaf desiccation and thus improving turgidity and plant growth (Abdelaal et al., 2021). Microbial inoculation improves plant ion flux and the synthesis and use of organic solutes for osmotic adjustments (Santander et al., 2017). In addition, microbial interactions improve stomatal regulation, leaf water use efficiency, and oxidative stress management (Paneque et al., 2016; Tak et al., 2021). Plants protect photosystem II (PSII) activity by regulating light harvesting mechanisms critical for biomass production (Saccon et al., 2022). Low water availability reduces the potential agricultural uses of arable land. However, soil and plants can be supplemented with beneficial bacteria to increase food production. The suitability of selected microbes depends on soil type, bacterial strain and concentration, plant species, and stress conditions. Plant microbiomes are integrated within the host into single units of evolution called holobionts. The seed endosphere is a little-investigated plant microhabitat, more recently receiving attention for its potential as a reservoir and vector of beneficial microbes (Berg and Raijmakers, 2018; Rahman et al., 2018). Seed endophytes have been detected in many crop plants, including cereals and legumes, and can improve plant growth and ecophysiological parameters (Alibrandi et al., 2018; Liu et al., 2020). In barley, seed endophytes increased plant biomass activities when used as inoculants (Rahman et al., 2018).

The two bacterial strains used in this work (Pantoea sp. “ITS group 2” and Pseudomonas sp. “ITS group 11”) were selected as best candidates among a series of new isolates from barley seeds (Rahman et al., 2018). They were demonstrated to be consistently associated to barley seed across a variety of cultivars and years (Rahman et al., 2018). Taxonomical identification was performed by 16S rRNA gene sequencing and the isolates were characterized at strain level by ITS polymorphism analysis. Due to their superior performance in barley growth promotion and biocontrol activity (Rahman et al., 2018), these two strains were chosen for the current study. Moreover, they showed ability to efficiently colonize barley roots upon seed germination (Rahman et al., 2018). This study investigates the potential of selected endophytes to improve biomass and physiology of barley under drought stress. The selection of bacterial strain and appropriate level in dry soil is the key component of this manuscript. Barley was selected due to its importance as a global staple food and the availability of preliminary data using seed endophytes as PGPB (Rahman et al., 2018). We tested the following hypotheses: 1) Bacterial inoculation enhances soil conditions (soil CO_2_ flux), barley growth, nutrient acquisition and plant survival under drought stress; 2) Water limitation improves stomatal resistance and photosynthesis by regulating antioxidiant defense in barley with bacterial inoculation.

## Material and methods

### Surface sterilization of seeds

Seeds were submerged in 70% ethyl alcohol (EtOH) for 5 minutes under gentle manual shaking before rinsing with sterile H_2_O for 5 minutes under manual shaking. Next, the seeds were immersed in a 1:1 mixture of Danklorix (~2.4% active NaClO) and disinfection solution (1 g Na_2_CO_3_, 30 g NaCl, 1.5 g NaOH per L distilled water) for 1 h at 25°C under mechanical shaking (90 rpm). Finally, the seeds were washed with sterile H_2_O for 10, 20, 30, 40, and 50 minutes at 25°C under mechanical shaking (90 rpm).

### Inoculation of surface-sterilized seeds (bio-priming)

There were four treatments: (1) uninoculated, (2) inoculated with *Pantoea* sp. (ITS Group 2), (3) inoculated with *Pseudomonas* sp. (ITS Group 11), and (4) inoculated with *E. coli.* For each treatment, 170 seeds were immersed in ~35 mL of the corresponding bacterial suspension at the following concentrations:


$$Pantoea=3.10\times 10^{7}\;CFU\;mL^{-1}$$

$$Pseudomonas=4.95\times 10^{7\;}\;CFU\;mL^{-1}$$

$$Escherichia\;coli=1.50\times 10^{7}\;CFU\;mL^{-1}$$

The bacterial suspensions were obtained by diluting an overnight liquid culture (medium: CASO Bouillon) with sterile 0.03 M MgSO_4_. Seeds of the uninoculated treatment were immersed in 35 mL of sterile 0.03 M MgSO_4_. Inoculation with *E. coli* was used as an additional control to account for the possible effects of adding organic biomass.

### Bacterial inoculation in soil and growth conditions

Twelve days after sowing of inoculated seed in soil, the pots were inoculated with 1 mL overnight liquid culture (~3 × 10^9^ CFU mL^–1^) of the corresponding bacterium. Pots of the uninoculated treatment were amended with 1 mL sterile CASO Bouillon. The experiment was conducted in a greenhouse under controlled environmental conditions (average temperature 25 ± 2°C, relative humidity 50%, 16/8 h (light/dark) photoperiod, average daily light integral 200–250 μmol m^–2^ s^–1^ which equals to Daily Light Integral (DLI) 14.40 mol m^-2^ d^-1^). Seedlings were at 12 days transplanted into plastic tubes (20 cm length, 5 cm diameter) containing sand (50%), clay (30%), and gravel (20%), with nutrients supplied as Wuxal Super (Aglukon, Düsseldorf, Germany) for 10 days.

The water-holding capacity (WHC) of the potted soil was determined by using the method of Veihmeyer and Hendrickso 1931 (cited in Abideen et al., 2020a). The 100% WHC was used as the reference point for cultivation. Plants at 50% WHC (showed chronic stress and associated acclimation responses. Therefore, the water holding capacity was maintained around 50% for drought treatment in this study as described in Abideen et al. (2020). All plants were irrigated twice a week with Hoagland’s nutrient solution (Epstein, 1972). Plants were harvested after stress at 28 days, with leaf water relations and gas exchange parameters measured before the final harvest.

### Soil water content, temperature and CO_2_ flux

Soil respiration was measured using an LI-8100 soil efflux chamber system (LI-COR Inc., Lincoln, USA) and a dark survey chamber (10 cm diameter) within 30 min after removing the plant tops from the pots. The survey chamber fitted onto the brims of the pots (Kammann et al., 2011). The offset of each pot (distance from the soil surface to the pot brim) was entered into the LI-8100 system software to calculate the correct system volume and soil CO_2_ efflux. Measurement time and observation delay were set to 60 and 20 s, respectively, to provide sufficient time for chamber volume mixing and CO_2_ release monitoring. The increase in CO_2_ concentration always exhibited a linear slope, with R^2^ > 0.99. This result validated the automatic calculation of CO_2_ flux using LI-8100 software using the ideal gas law and linear regression. The respiration value is the CO_2_ flux in µmol m^−2^ s^−1^. The soil water content and temperature were measured with the help of WET150 Multi-Parameter Soil Sensor.

### Growth parameters

Shoot and root fresh weights (FW) were recorded immediately after harvest using weighing balance. Shoot and root samples were oven-dried at 80°C for 48 h to determine dry weights. Some fresh samples were also frozen immediately in liquid nitrogen and stored at –20°C for antioxidiant enzyme assays. Leaf area (whole plant basis) was measured with a portable area meter (LI-COR-3000C). At least five biological replicates were used.

### Leaf gas exchange, chlorophyll and chlorophyll fluorescence

Leaf gas exchange parameters (net photosynthetic rate, respiration, stomatal conductance, intercellular CO_2_ concentration, transpiration rate, and water use efficiency (WUE) = net photosynthetic rate/stomatal conductance) were measured on fully expanded young leaves between 8 a.m to 4 pm. Steady state CO_2_/H_2_O leaf gas exchange readings were recorded using a LI-COR 6400XT photosynthesis system (LI-COR Inc., Lincoln, NE, USA) at 400 μmol mol^–1^ CO_2_ atmospheric concentrations, 30°C block temperature, ≤ 2 kPa vapor pressure deficit, and ~1,000 μmol m^–2^ s^–1^ light intensity. Estimated chlorophyll content was recorded with a SPAD 502 (Konica Minolta, Japan).

Chlorophyll fluorescence parameters were determined two days before plant harvest using a pulse modulated chlorophyll fluorescence meter (Junior PAM, Walz, Germany) on the same leaves used for CO_2_/H_2_O gas exchange measurements. Minimal (Fo) and maximal fluorescence (Fm) values were recorded on dark (25 min) adapted leaves to calculate the maximum photochemical quantum yield of PSII [(Fv/Fm = (Fm – Fo)/Fm)] according to the method of Kitajima and Butler (1975). Steady state (Fs), maximal (Fm′), and minimal fluorescence (Fo) were measured on light-adapted leaves. Effective photochemical quantum yield of PSII was calculated according to the formula [(Fm′ – Fs)/Fm′] as described by Genty et al. (1989). Non-photochemical quenching (NPQ) was calculated as NPQ = Fm′/(Fm′ – 1), formulated by Bilger and Bjorkman (1990). The electron transport rate (ETR) was calculated according to the formula described in Krall and Edwards (1992):


ETR=PSII×PPFD×0.5×0.84

where PPFD is leaf photosynthetic photon flux density, 0.5 is the factor used to assume an equal amount of energy distribution between two photosystems (PSII and PSI), and 0.84 is the factor used to assume leaf absorbance. The risk of oxidative stress was determined as ETR/A_gross_, as described in Salazar-Parra et al. (2012).

### Lipid peroxidation and enzyme assays

Malonyldialdehyde (MDA) levels was determined on fresh leaf as a damage (stress) marker using the method of Hernandez et al. (2001). The measurement of catalase (CAT, EC 1.11.1.6) activity was performed according to Aebi (1984). Ascorbate peroxidase (APX, EC 1.11.1.11) activity was determined Nakano & Asada, (1981). Activity of superoxide dismutase (SOD) was determined according to the method of Beyer and Fridovich, 1987. Glutathione reductase (GR, EC 1.6.4.2) activity was determined as performed by Foyer and Halliwell (1976). Guaiacol peroxidase (GPX, EC 1.11.17) activity was measured as described by Tatiana et al. (1999).

### Analysis of Na^+^, K^+^, Mg^2+^, Ca^2+^

Dried shoot and root samples (20 mg) were extracted with 10 mL HNO3 (0.5%) in a water bath (80°C) for 12 h. Na^+^, K^+^, Mg^2+^, and Ca^2+^ concentrations were determined using an atomic absorption spectrometer (AAS PE2100, Perkin Elmer, United States, MA 02451, Waltham, 940). The K^+^/Na^+^ ratio was calculated and used to indicate K+ and Na+ ion selectivity for absorption and transport (Pitman, 1965).

### Statistical analysis

Analysis of data (n = 5) was performed using SPSS (ver. 11) software, with significant differences among means (*P*< 0.05) assessed by Fisher’s protected least significance difference (LSD). The data were analyzed using two way analysis of variance (ANOVA) to identify significant effects, drought, bacteria and drought x bacteria interaction. of the experiment at P< 0.05. (**Table S1**).

## Results

### Soil water content, temperature, and CO_2_ flux

**Figure 1:**
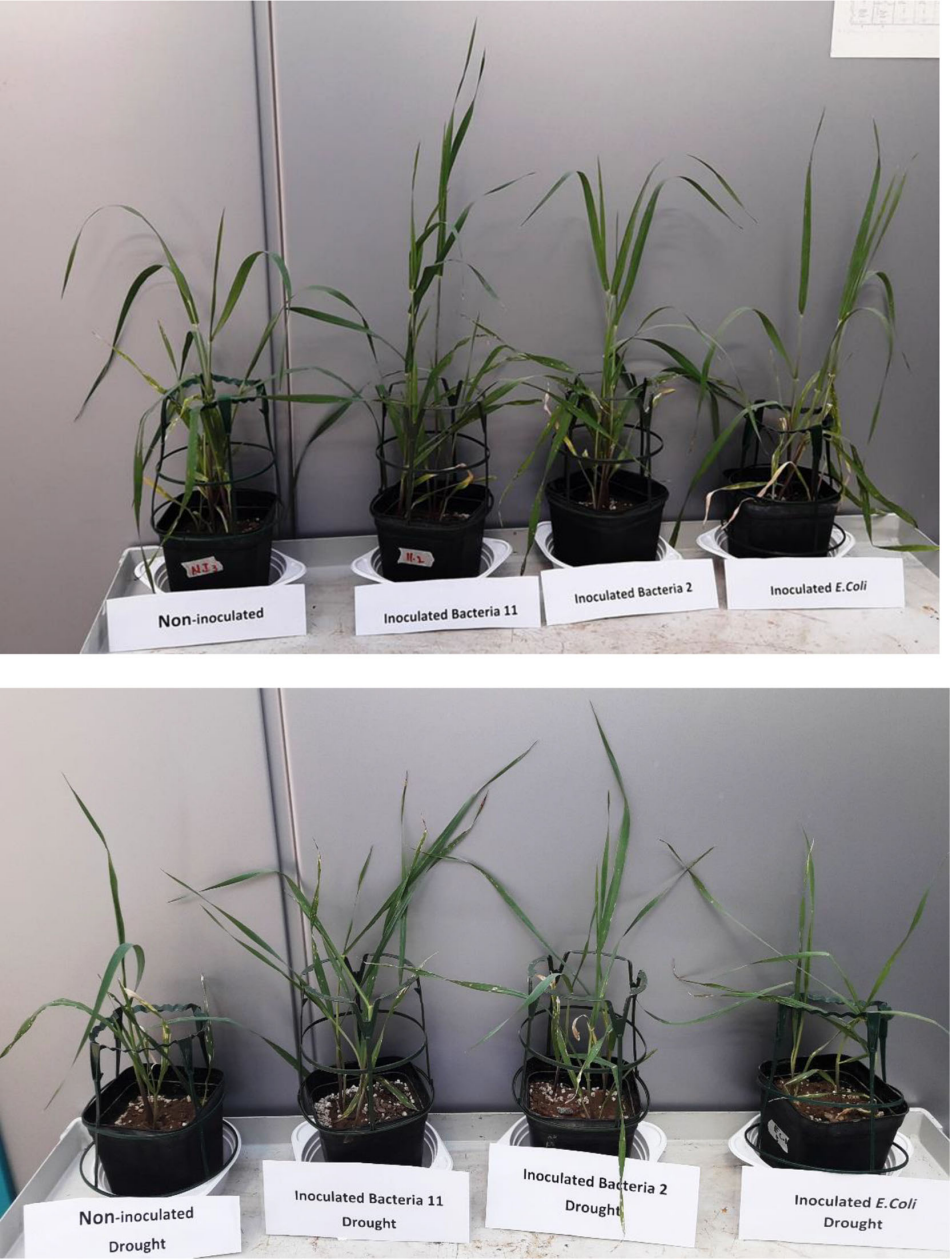
Shoot and root growth of drought-stressed and non-stressed (well-watered) *Hordeum vulgare* inoculated with three bacterial strains (*Pseudomonas* sp. (ITS Group 11), *Pantoea* sp. (ITS Group 2), or *E. coli*).

For soil data, two-way ANOVA showed a significant individual effect of both drought (D) and bacteria (B) but their interactions (D X B) The volumetric soil water content was monitored during the water deficit stress treatments. Soil inoculated with *Pantoea* displayed higher soil water contents under water deficit and well-watered conditions compared to control (no bacteria added). There were no change in soil temperature was observe in soil throughout the study regardless of the PGPR treatments. Inoculation with *Pseudomonas* or *Pantoea* both enhanced (about 2 folds) soil gas exchange (soil carbon flux) under water deficit and well-watered conditions than un-inoculated treatments (**Figure 1**).

### Plant growth

**Figure 2:**
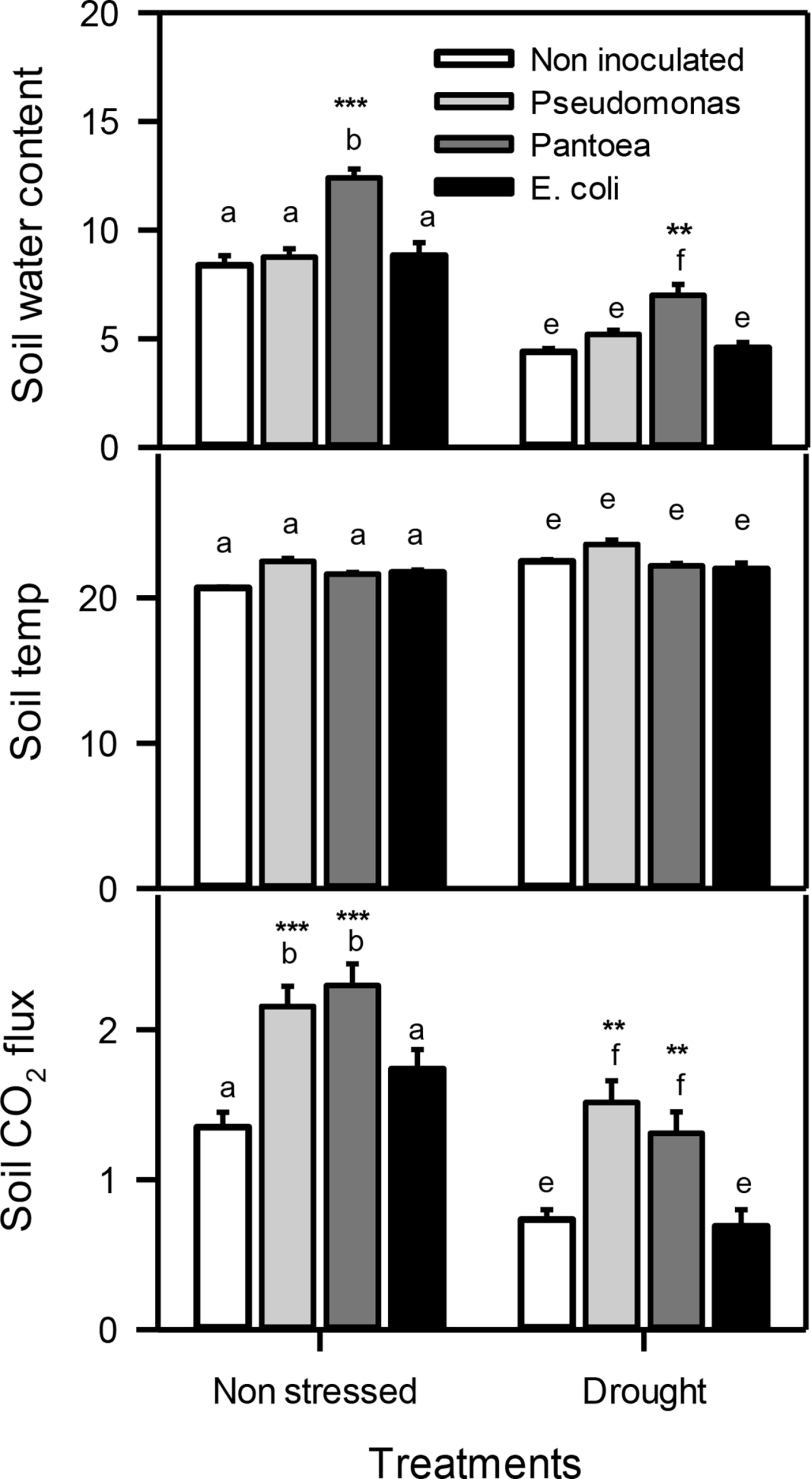
Soil water content, soil temperature (temp), and soil CO_2_ flux of drought-stressed and non-stressed (well-watered) *Hordeum vulgare* inoculated with three bacterial strains (*Pseudomonas* sp. (ITS Group 11), *Pantoea* sp. (ITS Group 2), or *E. coli*). F and P (***P< 0.001, **P< 0.01) values of the two-way ANOVAs are presented, drought, bacteria and drought x bacteria interaction. The lower case letters shows the significant differences among means (P< 0.05) assessed by Fisher’s protected least significance difference (LSD).

Plant total leaf fresh weight (FW) increased (23-29%) in non-stressed barley inoculated with *Pseudomonas* or *Pantoea* compared to other treatments. Water deficit caused a significant decreased in leaf FW, but the reduction was lower particularly in plants inoculated with *Pseudomonas*. Additionally, water deficit also reduced the stem and root FWs relative to well-watered plants. The inoculation of *Pseudomonas* or *Pantoea* under water deficit treatment caused a significant increase in the root length (1-2 folds) and 925%) leaf area compared to control plants (**Figure 2**).

### Leaf chlorophyll content, gas exchange, and chlorophyll fluorescence

**Figure 3:**
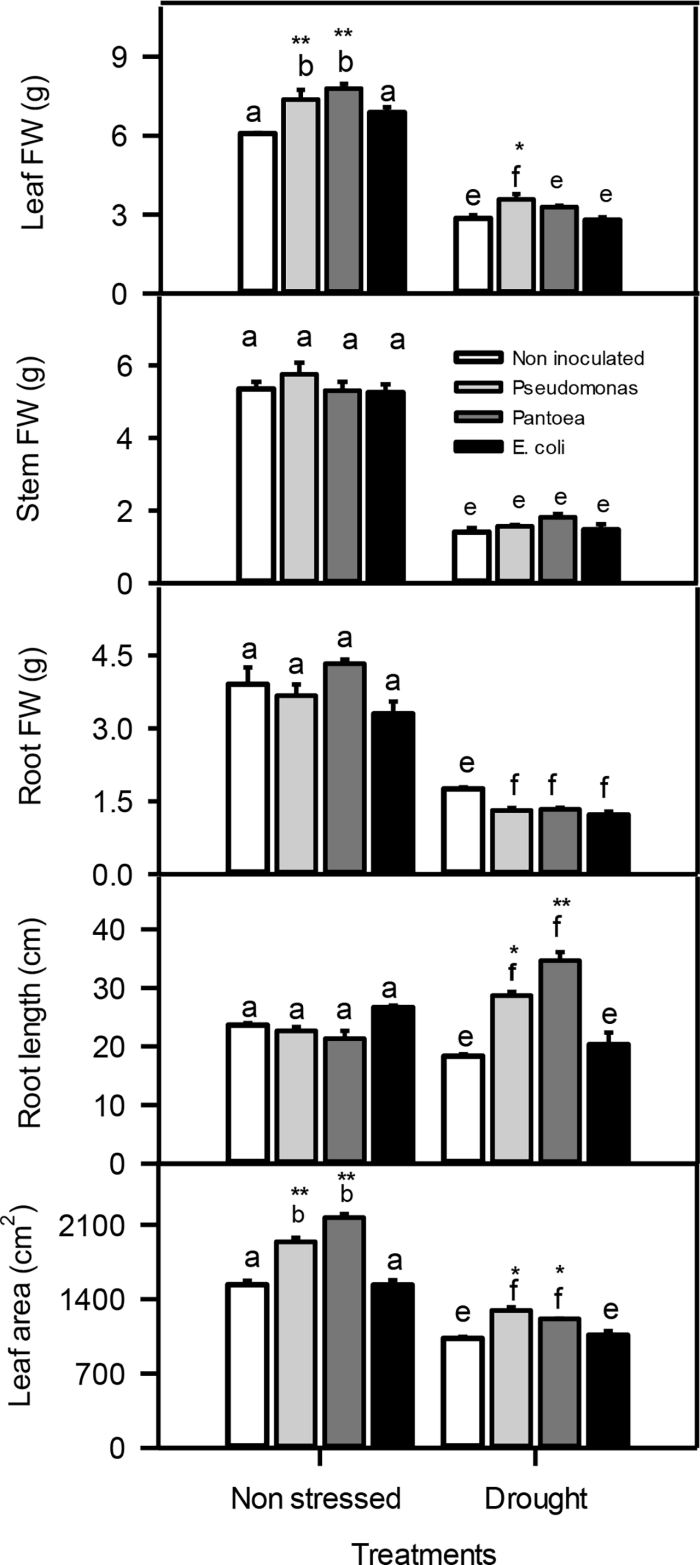
Leaf, stem, and root fresh weight (FW), root length, and leaf area of drought-stressed and non-stressed (well-watered) *Hordeum vulgare* inoculated with three bacterial strains (*Pseudomonas* sp. (ITS Group 11), *Pantoea* sp. (ITS Group 2), or *E. coli*). F and P (** P < 0.01, *P < 0.05) values of the two-way ANOVAs are presented, drought, bacteria and drought x bacteria interaction. The lower case letters shows the significant differences among means (P< 0.05) assessed by Fisher’s protected least significance difference (LSD).

About (18%) increase in total chlorophyll contents was detected in the well-watered and water-stressed plants inoculated with *Pseudomonas* or *Pantoea* than the control plants (**Figure 3**). *Pseudomonas* and *Pantoea* inoculation also increased net photosynthesis (33% in pentoa and 54% in *Pseudomonas*) while the reverse was true for *E. coli* (**Table 1**). Stomatal conductance increased about (44%) in drought-stressed plants inoculated with *Pseudomonas*. Internal CO_2_ concentration (Ci) decreased in well-watered plants inoculated with *Pseudomonas* or *Pantoea*, but in decline were prominent only with and water-stressed plants inoculated with *Pseudomonas* under drought. The Ci/Ca ratio decreased in well-watered plants inoculated with *Pseudomonas* but increased in water-stressed plants compare to other treatments (**Table 1**). Leaf transpiration increased about (27%) in water-stressed plants inoculated with *Pseudomonas* or *Pantoea* compare to other treatments (**Table 1**). Water use efficiency (WUE) increased in well-watered plants inoculated with *Pseudomonas* or *Pantoea* compare to control treatments, while rate of respiration rates increased in inoculated plants relative to uninoculated plants (**Table 1**).

**Table 1:** Photosynthesis, stomatal conductance, intercellular carbon dioxide (Ci) and ratio of intercellular CO_2_ with atmospheric CO_2_ (Ci/Ca), transpiration, water use efficiency (WUE), and respiration of drought-stressed and non-stressed (well-watered) *Hordeum vulgare* inoculated with three bacterial strains (*Pseudomonas* sp. (ITS Group 11), *Pantoea* sp. (ITS Group 2), or *E. coli*).

Treatments	Photosynthesis(μmol m^–2^ s^–1^)	Conductance(mol m^–2^s^–1^)	Intercellular CO_2_(μmol mol^–1^)	Ci/Ca ratio	Transpiration(mmol m^–2^ s^–1^)	WUE(μmol CO_2_ mol^-1^ H_2_O)	Respiration(μmol m^–2^ s^–1^)	Chlorophyll(SPAD)
**Non-stressed**								
Uninoculated	12.13 ± 0.19a	0.13 ± 0.005a	236.74 ± 5.69b	0.60 ± 0.01b	1.71 ± 0.08b	7.11 ± 0.33a	0.81 ± 0.07a	39.01 ±1.24a
*Pseudomonas*	14.80 ± 0.42b*	0.14 ± 0.006b	213.44 ± 11.76a**	0.55 ± 0.02a	1.56 ± 0.14b**	9.76 ± 1.05b*	1.29 ± 0.19b**	45.46 ± 1.08b*
*Pantoea*	14.14 ± 0.58b*	0.14 ± 0.005b	225.52 ± 1.67a*	0.58 ± 0.01ab	1.53 ± 0.05b	9.24 ± 0.35b*	1.63 ± 0.09b**	43.34 ± 1.56b*
*E. coli*	10.51 ± 0.27a	0.11 ± 0.004a	239.40 ± 10.30b	0.61 ± 0.02b	1.20 ± 0.05a	8.79 ± 0.68a	1.33 ± 0.10b	35.28 ± 1.02a
**Drought-stressed**								
Uninoculated	9.25 ± 0.45e	0.09 ± 0.003e	213.53 ± 10.55f	0.54 ± 0.02e	1.08 ± 0.06e	9.13 ± 0.72e	1.07 ± 0.06e	38.88 ± 0.97e
*Pseudomonas*	14.26 ± 0.61f*	0.13 ± 0.006f*	204.71 ± 3.43e	0.57 ± 0.02f	1.60 ± 0.07f**	8.92 ± 0.26e*	1.36 ± 0.10f*	45.96 ± 0.54f*
*Pantoea*	12.79 ± 0.38f	0.12 ± 0.006f*	216.24 ± 4.34f*	0.59 ± 0.03f	1.54 ± 0.07f**	8.32 ± 0.17e*	1.21 ± 0.04f*	43.38 ± 0.69f*
*E. coli*	10.15 ± 0.18e	0.10 ± 0.003e	219.75 ± 3.74f	0.55 ± 0.01e	1.28 ± 0.07e	8.14 ± 0.35e	1.23 ± 0.12f	35.68 ± 0.75e

F and P (** P< 0.01, *P< 0.05, ns = non-significant) values of the two-way ANOVAs are presented, drought, bacteria and drought x bacteria interaction. Different lower-case letters within a column significantly differ at P ≤ 0.05.

Bacterial inoculation did not change the potential quantum yield of PSII (Fv/Fm) under well-watered or water deficit conditions (**Table 2**). ETR did not change in well-watered plants inoculated with *Pseudomonas* or *Pantoea* but increased in plants inoculated with *Pantoea* relative to uninoculated plants under drought. NPQ increased in well-watered and water-stressed plants inoculated with *Pseudomonas* or *Pantoea* (**Table 2**). Photoinhibition and ETR/A ratios decreased (photoinhibition 91% and 49% ETR/A) in barley plants inoculated with *Pseudomonas* or *Pantoea* compared to control under well-watered and water-deficit conditions (**Table 2**).

**Table 2:** Chlorophyll florescence parameters (photochemical efficiency of photosystem II [Y (II)], electron transport rate (ETR), non-photochemical quenching (NPQ), maximum photosynthetic efficiency of PSII (Fv/Fm), photoinhibition and oxidative stress (ETR/A)) of drought-stressed and non-stressed (well-watered) *Hordeum vulgare* inoculated with three bacterial strains (*Pseudomonas* sp. (ITS Group 11), *Pantoea* sp. (ITS Group 2), or *E. coli*).

Treatments	Y (II)	ETR	NPQ	Fv/Fm	Photoinhibition	ETR/A
**Non-stressed**						
Uninoculated	0.66 ± 0.003a	79.46 ± 0.33a	0.33 ± 0.02a	0.79 ± 0.00a	1.65 ± 0.24a	6.84 ± 0.38a
*Pseudomonas*	0.66 ± 0.002a	79.80 ± 0.23a	0.36 ± 0.04a	0.82 ± 0.01a	0.79 ± 0.15b**	5.38 ± 0.39b**
*Pantoea*	0.65 ± 0.007a	77.63 ± 0.86a	0.39 ± 0.01b*	0.82 ± 0.00a	0.80 ± 0.38b**	5.48 ± 0.47b**
*E. coli*	0.65 ± 0.004a	78.71 ± 0.55a	0.39 ± 0.00b	0.81 ± 0.01a	2.27 ± 0.23a	7.48 ± 0.49a
**Drought-stressed**						
Uninoculated	0.65 ± 0.002e	78.80 ± 0.30e	0.26 ± 0.02e	0.80 ± 0.04e	3.60 ± 0.06e	8.07 ± 1.25f
*Pseudomonas*	0.64 ± 0.001e	77.41 ± 0.20e	0.34 ± 0.01f*	0.80 ± 0.06e	1.88 ± 0.29f**	5.42 ± 1.45e**
*Pantoea*	0.67 ± 0.005e	80.39 ± 0.68f	0.32 ± 0.02f*	0.79 ± 0.08e	1.31 ± 0.16f**	6.27 ± 1.89e**
*E. coli*	0.64 ± 0.009e	76.77 ± 1.15e	0.28 ± 0.00e	0.76 ± 0.02e	5.02 ± 0.80e	7.40 ± 2.51f

F and P (0.001, **P < 0.01, *< 0.05, ns = non-significant) values of the two-way ANOVAs are presented, drought, bacteria and drought x bacteria interaction. Different lower-case letters within a column significantly differ at P ≤ 0.05.

### Antioxidant enzymes

**Figure 4:**
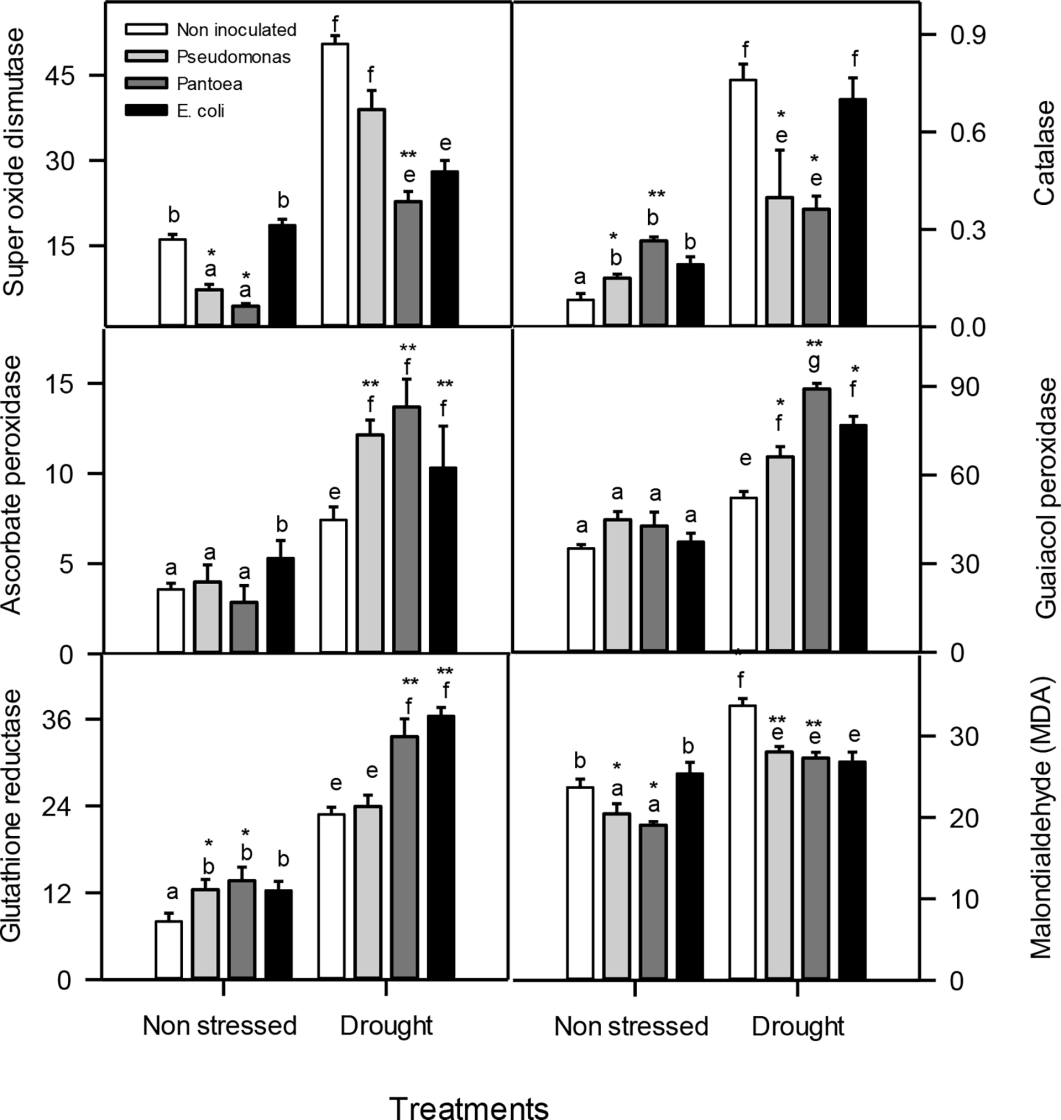
Antioxidant enzyme levels (μmol mg-1 prot min-1) FW—superoxide dismutase (SOD), catalase (CAT), ascorbate peroxidase (APX), guaiacol peroxidase (GPOX), glutathione reductase (GR), and malondealdehyde (MDA (μmol g-1 FW))—in drought-stressed and non-stressed (well-watered) Hordeum vulgare inoculated with three bacterial strains (*Pseudomonas sp*. (ITS Group 11), Pantoea sp. (ITS Group 2), or *E. coli*). F and P (** P< 0.01, *P< 0.05) values of the two-way ANOVAs are presented, drought, bacteria and drought x.The lower case letters shows the significant differences among means (P< 0.05) assessed by Fisher’s protected least significance difference (LSD).

Well-watered and drought-stress inoculated plants accumulated lower SOD enzyme activities than uninoculated plants. Plants inoculated with *Pseudomonas* or *Pantoea* enhanced CAT activities under well-watered conditions and decreased CAT activities under drought stress than uninoculated plants (**Figure 4**). Plants inoculated with *Pseudomonas* or *Pantoea* had higher APX enzyme activities under drought stress relative to uninoculated plants. Plants inoculated with *Pseudomonas* or *Pantoea* had higher GPX activities under well-watered conditions relative to uninoculated plants (**Figure 4**). Well-watered and drought-stressed inoculated plants improved GR activities than uninoculated plants(**Figure 4**). Interestingly, MDA contents decreased in well-watered and drought-stressed plants inoculated with *Pseudomonas* or *Pantoea* (**Figure 4**).

### Minerals analysis

Shoot Ca^2+^ increased in well-watered plants inoculated with *Pseudomonas* or *Pantoea* compared to other treatments. Root Ca^2+^ increased in well-watered plants inoculated with *Pseudomonas* or *Pantoea* and water-stressed plants except *Pseudomonas* in water-stressed plants (**Table 3**). Shoot Mg^2+^ levels enhanced in well-watered and drought-stressed plants inoculated with *Pseudomonas* and *Pantoea*. Root Mg^2+^ increased in well-watered plants inoculated with *Pseudomonas* but did not change under drought stress (**Table 3**). Shoot and root K^+^ and the shoot and root K^+^/Na^+^ ratios increased in well-watered and drought-stressed plants inoculated with *Pseudomonas* and *Pantoea*, relative to control treatments (**Table 3**). Shoot Na^+^ increased in well-watered plants inoculated with *Pantoae* but decreased with plants inoculated with *Pseudomonas*. Shoot Na^+^ increased in drought-stressed plants inoculated with *Pantoea, Pseudomonas* relative to uninoculated plants. Well-watered inoculated plants accumulated higher root Na^+^ than uninoculated plants compared to control (**Table 3**). Plants under water deficit had higher root Na^+^ than well-watered plants, particularly those inoculated with *Pseudomonas*. (**Table 3**).

**Table 3:** Shoot and root cation (Ca^++^, Mg^++^, K^+^, Na^+^) concentrations (mmol kg^–1^ FW) and shoot K^+^/Na^+^ ratio of drought-stressed and non-stressed (well-watered) *Hordeum vulgare* inoculated with three bacterial strains (*Pseudomonas* sp. (ITS Group 11), *Pantoea* sp. (ITS Group 2), or *E. coli*).

Treatments	Shoot Ca^++^	Shoot Mg^++^	Shoot K^+^	Shoot Na^+^	Shoot K^+^/Na^+^
Non-stressed					
Uninoculated	0.58 ± 0.02a	13.23 ± 0.64a	56.38 ± 0.40a	17.093 ± 0.43b	3.30 ± 0.06a
*Pseudomonas*	0.71 ± 0.02b*	15.94 ± 0.87b	65.11 ± 0.87b*	13.636 ± 2.06a	5.11 ± 0.71b*
*Pantoea*	1.26 ±0.09b*	19.85 ± 0.30c	82.48 ± 2.70c*	21.813 ± 0.47c*	3.79 ± 0.21a
*E. coli*	0.85 ± 0.05a	17.04 ± 0.02b	68.55 ± 1.17b	18.952 ± 0.70b	3.63 ± 0.12a
Drought-stressed					
Uninoculated	4.03 ± 0.23e	61.76 ± 2.33e	296.45 ± 13.98e	97.24 ± 8.22e	3.08 ± 0.16e
*Pseudomonas*	4.85 ± 0.09f	69.36 ± 1.41f*	448.68 ± 12.15f*	123.04 ± 8.17f*	3.70 ± 0.28f*
*Pantoea*	4.61 ± 0.14e	62.21 ± 1.71e	350.04 ± 10.01f*	117.75 v 7.91f	3.01 ± 0.24e
*E. coli*	4.65 ± 0.10e	64.10 ± 1.69e	419.55 ± 14.75f	125.80 ± 9.23f	3.36 ± 0.17e
	Root Ca^++^	Root Mg^++^	Root K^+^	Root Na^+^	Root K^+^/Na^+^
Non-stressed					
Uninoculated	1.68 ± 0.10a	6.57 ± 0.15a	55.41 ± 2.67a	290.88 ± 25.90 a	1.93 ± 0.09b
*Pseudomonas*	2.55 ± 0.13b*	8.32 ± 0.15b	66.70 ± 1.89b	331.51 ± 22.50 b*	1.46 ± 0.13a*
*Pantoea*	2.29 ± 0.01b*	6.71 ± 0.22a	89.59 ± 5.63b*	306.38 ± 26.56b*	2.01 ± 0.25b
*E. coli*	1.80 ± 0.05a	6.36 ± 0.23a	45.78 ± 1.61a	388.67 ± 16.70b	1.18 ± 0.05a
Drought-stressed					
Uninoculated	6.80 ± 0.43f	11.34 ± 0.58e	63.93 ± 2.36e	375.50 ± 24.24f	1.44 ± 0.13e
*Pseudomonas*	4.46 ± 0.16e	11.02 ± 0.36e	112.27 ± 4.60g*	421.79 ± 49.66g*	1.98 ± 0.25f*
*Pantoea*	9.95 ± 0.64g	12.12 ± 0.58f	79.70 ± 2.07f	330.45 ± 8.47e	1.51 ± 0.08e
*E. coli*	7.51 ± 0.32f	11.44 ± 0.27e	63.44 ± 4.72e	398.41 ± 15.29f	1.32 ± 0.08e

F and P (*P < 0.05, ns = non-significant) values of the two-way ANOVAs are presented, drought, bacteria and drought x bacteria interaction. Different lower-case letters within a shoot or root column significantly differ at P ≤ 0.

## Discussions

Drought is a major abiotic factor that inhibits crop yields but association of seed-associated bacterial endophytes of *Hordeum vulgare* are beneficial for rhizosphere soil health and its proper application, relieved the adverse effects of water deficit on barley grown under water limited areas to ensure productivity of such an important food crops (Chandra et al., 2021; Tyagi and Pandey, 2022; Kour and Yadav, 2022). In this study, bacterial inoculation enhanced both soil carbon flux and soil moisture under drought stress, regulating the ecophysiological performance (growth, net photosynthesis, and mineral acquisition) of barley seedlings. Stimulation of soil carbon flux due to *Pseudomonas* and *Pantoea* inoculation in dry arid areas in barley was might be associated with increased soil respiration that triggers higher microbial activity. Higher soil metabolic output with microbial inoculation indicates that barley cultivation with PGPB is a suitable strategy for enhancing carbon sequestration and thus contributing to climate change mitigation (Radicetti et al., 2020). Improvement of soil parameters especially soil water acquisition (especially *Pantoea* treatment) under stressed and non-stressed conditions was also as reported for maize (Naseem and Bano, 2014; Goudarzi et al., 2023), which has been associated with exopolysaccharides (EPS) production. EPS significantly enhance plant growth (Naseem and Bano, 2014; Zayed et al., 2022) by colonizing plant roots, forming hydrophilic biofilms, and providing plant immune response (Sun et al., 2022). In addition, PGPB= use several mechanisms to improve plant growth such as maintaining sufficient nutrient supply or regulating hormone levels (Forni et al., 2017; Siddiqui et al., 2021). In this study, the introduced bacterial endophytes *Pseudomonas* and *Pantoea* emerged as mediators for enhancing total foliage biomass, as reported in *Capsicum annuum* (**Figure 2**) (Datta et al., 2011; Lin et al., 2020). Application of microbes can improve fruit quality by upregulating nitrogen metabolism and producing specific hormones that trigger water and mineral balance and promote belowground biomass (Ahemad and Kibret, 2014). In the present study, *Pseudomonas* (under drought and control both condition) and *Pantoea* inoculations (well water condition) increased leaf fresh biomass and other growth parameters which was reported earlier in maize under water deficit condition (Jeong et al., 2021). Plants inoculated with *Pseudomonas* or *Pantoea* improved plant root elongation under water deficit compared to the other treatments. Higher root production under drought suggests that barley seeds benefit from soil microbe/plant interactions with *Pseudomonas* and *Pantoea* endophytes to access optimum water and nutrient which is critical for biomass production (Lin et al., 2020; Verma et al., 2021). Increased root development from microbial amendments can also support seedling emergence and long-term survival of barley in poorly degraded, dry areas that appear futile for agriculture and thus improve sustainable agriculture to avoid food insecurity (Calvo et al., 2017; Abdelfadil et al., 2022). In addition, it was reported that PGPB strains enhance phytohormone production and other signals to modify root system architecture, such as increased lateral root branching and root hair development (Siddiqui et al., 2022). The proper root modification stimulated the leaf development that favors the photosynthesis and the activity of photochemical reaction. Chlorophyll is the main photosynthetic pigment that was stimulated due to PGPB application in barley under drought-stressed and well-watered conditions due to increased nutrient acquisition, as reported elsewhere (Dawwam et al., 2013; Dao et al., 2020). Enhanced synthesis of chlorophyll pigments and their accessory components improve photosynthetic rate as well asPSII efficiency and the protein-pigment complex function. Plants inoculated with PGPR developed drought tolerance, suggesting that plants treated with bacterial strains enhance CO_2_ assimilation and reduce water release by leaves (Armada et al., 2015; Mehrasa et al., 2022). In addition, under suboptimum conditions, plants release some photosynthetic assimilates as root exudates, which helps maintain bacterial colonization in the root zone, promoting mutual benefits such as increased plant resistance against abiotic stress (Samaniego-Gámez et al., 2016). It was reported that PGPR elevate photosynthesis in in plants by regulating endogenous sugar/abscisic acid signaling (Sati et al., 2021). In the present study, plants inoculated with *Pseudomonas* and *Pantoea* increased gas exchange, respiration, stomatal conductance and leaf transpiration under drought-stressed and well-watered conditions. However, they only increased WUE under well-watered conditions (**Table 3**), indicating that bacterial-inoculated plants improve plant growth by enhancing photosynthetic performance (Chandra et al., 2021). It was reported that bacterial strains in the root zone synthesize auxins (indole-3-acetic acid/indole acetic acid/IAA) that increase tissue cell division, photosynthetic pigment synthesis, and photosynthesis (Ahemad and Kibret, 2014; Mitra et al., 2022). In tobacco, it was established that CO_2_ produced in roots was transported to shoots for photosynthesis *via* the vascular system instead of stomata (Andrade et al., 2022). It was suggested that endophyte colonization changed the host plant’s photosynthetic apparatus, increasing the activity of light harvesting complexes and enhancing photosynthetic performance (Chaturvedi et al., 2022). Similarly, Liu et al. (2021) indicated that seed endophytes stimulate PSII efficiency in plants.

Bacterial inoculation of pepper plants increased ETR and NPQ which could be a consequence of the positive effect of PGPB (Samaniego-Gámez et al., 2016). Further, NPQ helped minimize the over-synthesis of O_2_ in PSII antenna, increasing NPQ in plants inoculated with bacterial strains to reduce excess light energy (Radhakrishnan and Baek, 2017). In our study, bacterial inoculation increased Fv/Fm under well-watered conditions but increased ETR and NPQ under drought stress. Interestingly, drought stress produced higher photoinhibition and ETR/A ratios than well-watered conditions but were substantially lower in plants inoculated with *Pseudomonas* and *Pantoea* than the other treatments. ETR increases due to high oxidation of the quinone acceptor (Qa) and its excitation energy, reducing oxidative damage (Garcia-Caparros et al., 2021). Yang et al. (2017) reported that PGPR provoke systemic tolerance of plants during abiotic stress (salt and drought). Abiotic stresses such as drought increase ROS formation, causing oxidative stress (Chiappero et al., 2019). Increased ROS production affects plants due to the oxidation of photosynthetic pigments in membrane lipids, proteins, and nucleic acids (Jajic et al., 2015; Mukarram et al., 2021). The upregulation of antioxidant enzymes, such as SOD and APX, is a significant plant response to drought Mukarram et al., 2021). Increased CAT, GR, and APX activities were reported in drought-stressed *Ocimun basilicum* inoculated with PGPR (Chiappero et al., 2019). In the present study, drought stress increased SOD, CAT, APX, GPX, and GR activities, and MDA content. Drought stress and bacterial inoculation combined reduced ROS production, as indicated by the decreased SOD activity and MDA content and increased APX, GPX, and GR activities.

Leaf growth and metabolite production are important parameters under water deficiency (Zulfiqar et al., 2020). In the present study, leaf area increased under drought-stressed and well-watered conditions, which may be linked to P uptake triggered by *Pseudomonas* or *Pantoea* inoculation, as reported for maize (Chaudhary et al., 2022). Microbial inoculum improves nutritional assimilation (N, P and K contents) in plants relative to uninoculated plants, possibly because the soil microbes compensate for nutrient deficiency, enhancing plant growth in nutrient-deficient environments (Bargaz et al., 2018). In the present study, *Pseudomonas* inoculation under well-watered conditions increased shoot and root K^+^ concentration but decreased shoot Na^+^ concentration. Sequestration of Na^+^ in roots and higher uptake of K^+^ in leaves increased the shoot K^+^/Na^+^ ratio in well-watered plants inoculated with *Pseudomonas*, as reported elsewhere for maize (Shahzad et al., 2022). Similarly, under water deficit, *Pseudomonas* preferentially increased shoot K+, retained root Na^+^, and enhanced the shott K^+^/Na^+^ ratio relative to the other treatments. De Inoculation of *Pseudomonas* increased seedling growth in low fertile soil by compensating for nutrient deficiency through the synthesis of plant growth-promoting hormones at the root interface, stimulating root development and increasing soil water and nutrient absorption (Amora-Lazcano et al., 2022; Mehrasa et al., 2022).

In addition, Ca^2+^ is critical for plant growth, playing an important role in cell wall and membrane development, photosynthesis and ion homeostasis and acting as a signaling molecule in the cytosol (Shabbir et al., 2022). Recently, Ahmed et al. (2021) showed that Ca^2+^ acts as a signaling agent, enhancing plant stress resistance in unfavorable environmental conditions. In the present study, plant Ca^2+^ concentration increased substantially in bacterial-treated plants compared to uninoculated plants and even under drought stress. In addition to Ca^2+^, Mg^2+^ plays an important role in carbohydrate partitioning, CO_2_ fixation during photosynthesis, and reactive oxygen species (ROS) formation (Tewari et al., 2021). Mg^2+^ increases root surface area and overall root growth, enhancing photosynthetic assimilate synthesis and transport and carbohydrate translocation, alleviating drought stress (Alrashidi et al., 2022). In the present study, shoot Mg^2+^ increased in drought-stressed plants inoculated with *Pseudomonas* while root Mg^2+^ did not change. Well-watered inoculated plants increased shoot Mg^2+^ relative to uninoculated plants, particularly in plants inoculated with *Pantoea*. Well-watered plants inoculated with *Pseudomonas* increased root Mg^2+^ relative to uninoculated plants.

## Conclusions

Our results suggest that the appropriate selection of endophytes and their respective response is important for inducing drought stress resistance in barley. *Pseudomonas* and *Pantoea* inoculations improved growth, metal acquisition, photosynthesis, and oxidative stress tolerance in drought-stressed barley. The improved biomass production and crop yield with endophytic bacterial inoculation could be a solution for growing barley on poorly degraded lands. Further research is needed to confirm our findings under field conditions in saline and waterlogged areas to unlock the full potential of PGPB on crop performance.

## Data availability statement

The raw data supporting the conclusions of this article will be made available by the authors, without undue reservation.

## Author contributions

ZA, MC and SR: Conceptualization, Investigation. ZA and SR: Formal analysis, Methodology, Writing- original draft. MC, FaZ, H-WK, KH, FeZ and KHMS: Supervision, Conceptualization, Resources, Writing – review and editing, Funding acquisition. WD and FaZ: Formal analysis. All authors contributed to the article and approved the submitted version.

## Acknowledgments

This work was supported by the DAAD (The German Academic Exchange Service) Fund (grant P21067). The authors also acknowledge Taif University Researchers Supporting Project number (TURSP-2020/94), Taif University, Taif, Saudi Arabia.

## Conflict of interest

The authors declare that the research was conducted in the absence of any commercial or financial relationships that could be construed as a potential conflict of interest.

## Publisher’s note

All claims expressed in this article are solely those of the authors and do not necessarily represent those of their affiliated organizations, or those of the publisher, the editors and the reviewers. Any product that may be evaluated in this article, or claim that may be made by its manufacturer, is not guaranteed or endorsed by the publisher.
