# Supplementary material for: Seed Endophyte bacteria enhance drought stress tolerance in Hordeum vulgare by regulating, physiological characteristics, antioxidants and minerals uptake
Source: Front Plant Sci. 2022 Oct 5;13:980046. doi: 10.3389/fpls.2022.980046 (PMC9581713; doi:10.3389/fpls.2022.980046)
Supplement: Supplementary file 1 [file DataSheet_1.pdf]

Supplementary material

Table 1. The data were analyzed using two way analysis of variance (ANOVA) to identify significant effects, drought, bacteria and drought x bacteria interaction of the experiment at P < 0.05.

**Parameters**	**Source**	**Type III Sum Squares**	**df**	**Mean Square**	**F**	**Sig.**
Soil CO2 flux	Bacteria	4315	3	1438	27255	0
	drought	5136	1	5136	97315	0
	Bacteria * drought	0.594	3	0.198	3749	0.024
Soil water content	Bacteria	75136	3	25045	49199	0
	drought	153563	1	153563	301658	0
	Bacteria * drought	6981	3	2327	4571	0.011
Root fresh weight	drought	38557	1	38557	361278	0
	bacteria	1412	3	0.471	4411	0.019
	drought * bacteria	0.479	3	0.16	1497	0.253
Leaf fresh weight	drought	92003	1	92003	860708	0
	bacteria	4630	3	1543	14440	0
	drought * bacteria	1438	3	0.479	4484	0.018
	drought	91299	1	91299	786241	0
Stem fresh weight	bacteria	0.565	3	0.188	1622	0.224
	drought * bacteria	0.492	3	0.164	1411	0.276
	drought	759375	1	759375	137030	0
Root length	bacteria	605458	3	201819	36419	0
	drought * bacteria	418458	3	139486	25170	0
	drought	2.49E+09	1	2.49E+09	776762	0
Leaf area	bacteria	8.1E+08	3	2.7E+08	84096	0
	drought * bacteria	2.14E+08	3	71473264	22259	0
Photosynthesi+	Bacteria	107134	3	35711	59857	0
	drought	6294	1	6294	10550	0.003
	Bacteria * drought	7576	3	2525	4233	0.015
Stomatal Conductance	Bacteria	0.006	3	0.002	18249	0
	drought	0.003	1	0.003	28517	0
	Bacteria * drought	0.001	3	0	4039	0.019
Internal carbon dioxide	Bacteria	1458402	3	486134	2478	0.086
	drought	1352187	1	1352187	6893	0.015
	Bacteria * drought	403162	3	134387	0.685	0.57
Transpiration	Bacteria	0.689	3	0.23	9018	0
	drought	0.076	1	0.076	2992	0.097
	Bacteria * drought	0.779	3	0.26	10192	0
Water use efficiency	Bacteria	7498	3	2499	2147	0.121
	drought	0.013	1	0.013	0.011	0.916
	Bacteria * drought	11281	3	3760	3229	0.04
Respiration	Bacteria	1157	3	0.386	9173	0
	drought	0.002	1	0.002	0.055	0.817
	Bacteria * drought	0.517	3	0.172	4102	0.017

Non photochemical quenching	bacteria	0.014	3	0.005	3330	0.046
	drought	0.023	1	0.023	16409	0.001
	bacteria * drought	0.009	3	0.003	2168	0.132
Catalase	Drought	0.901	1	0.901	602163	0
	bacteria	0.171	3	0.057	38001	0
	Drought * bacteria	0.378	3	0.126	84150	0
Ascorbate peroxidase	Drought	331801	1	331801	514740	0
	bacteria	42866	3	14289	22167	0
	Drought * bacteria	43520	3	14507	22505	0
Glutathione reductase	Drought	1835226	1	1835226	881809	0
	bacteria	340798	3	113599	54583	0
	Drought * bacteria	139542	3	46514	22349	0
Guaiacol peroxidase	Drought	5946446	1	5946446	1171113	0
	bacteria	1773652	3	591217	116436	0
	Drought * bacteria	768012	3	256004	50418	0
Shoot Potassium	Drought	1128955	1	1128955	34459	0
	bacteria	6312647	3	2104216	64227	0
	Drought * bacteria	2716037	3	905346	27634	0
	Error	524194	16	32762		
Shoot Calcium	Drought	167475	1	167475	451900	0
	bacteria	22425	3	7475	20170	0
	Drought * bacteria	28428	3	9476	25569	0
Shoot Magnesium	Drought	14648563	1	14648563	3271286	0
	bacteria	83965	3	27988	6250	0.005
	Drought * bacteria	99692	3	33231	7421	0.002
